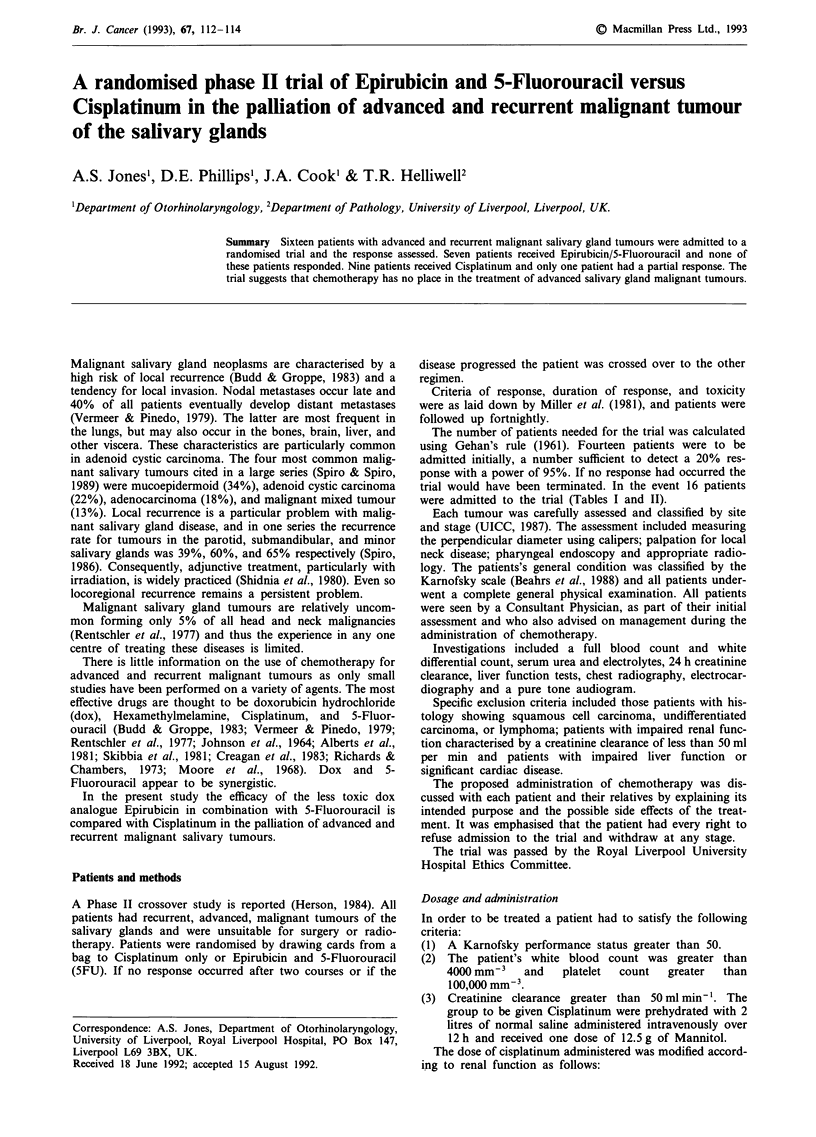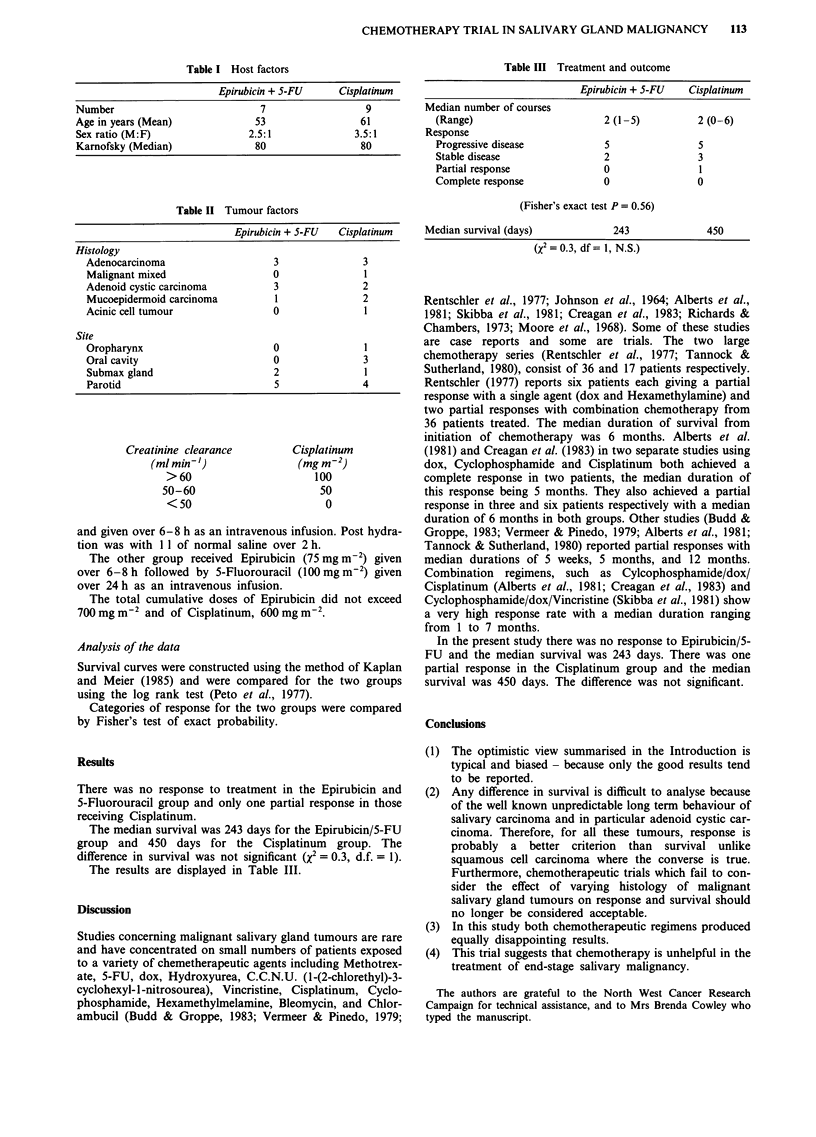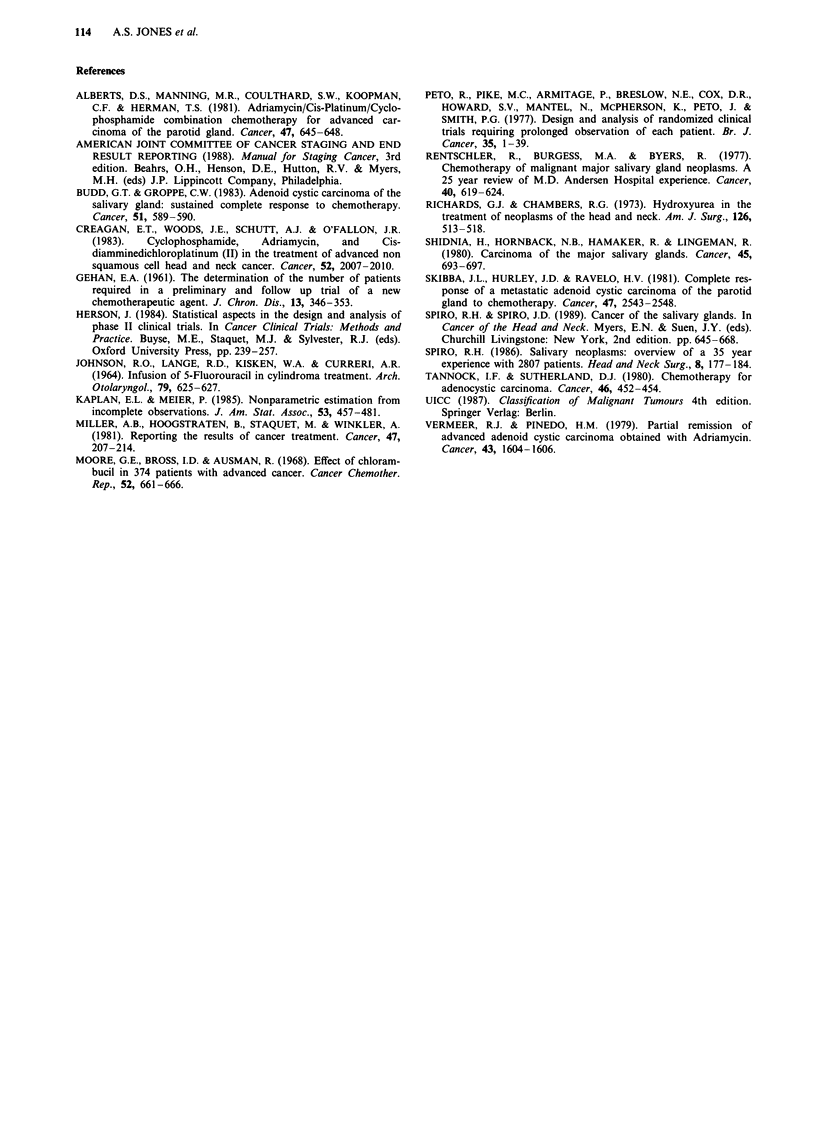# A randomised phase II trial of epirubicin and 5-fluorouracil versus cisplatinum in the palliation of advanced and recurrent malignant tumour of the salivary glands.

**DOI:** 10.1038/bjc.1993.19

**Published:** 1993-01

**Authors:** A. S. Jones, D. E. Phillips, J. A. Cook, T. R. Helliwell

**Affiliations:** Department of Otorhinolaryngology, University of Liverpool, UK.

## Abstract

Sixteen patients with advanced and recurrent malignant salivary gland tumours were admitted to a randomised trial and the response assessed. Seven patients received Epirubicin/5-Fluorouracil and none of these patients responded. Nine patients received Cisplatinum and only one patient had a partial response. The trial suggests that chemotherapy has no place in the treatment of advanced salivary gland malignant tumours.


Br. J. Cancer (1993), 67, 112–114 © Macmillan Press Ltd., 1993

### A randomised phase II trial of Epirubicin and 5-Fluorouracil versus Cisplatinum in the palliation of advanced and recurrent malignant tumour of the salivary glands

A.S. Jones[1], D.E. Phillips[1], J.A. Cook[1] & T.R. Helliwell[2]

[1]Department of Otorhinolaryngology, [2]Department of Pathology, University of Liverpool, Liverpool, UK.

**Summary** Sixteen patients with advanced and recurrent malignant salivary gland tumours were admitted to a randomised trial and the response assessed. Seven patients received Epirubicin/5-Fluorouracil and none of these patients responded. Nine patients received Cisplatinum and only one patient had a partial response. The trial suggests that chemotherapy has no place in the treatment of advanced salivary gland malignant tumours.

Malignant salivary gland neoplasms are characterised by a high risk of local recurrence (Budd & Groppe, 1983) and a tendency for local invasion. Nodal metastases occur late and 40% of all patients eventually develop distant metastases (Vermeer & Pinedo, 1979). The latter are most frequent in the lungs, but may also occur in the bones, brain, liver, and other viscera. These characteristics are particularly common in adenoid cystic carcinoma. The four most common malignant salivary tumours cited in a large series (Spiro & Spiro, 1989) were mucoepidermoid (34%), adenoid cystic carcinoma (22%), adenocarcinoma (18%), and malignant mixed tumour (13%). Local recurrence is a particular problem with malignant salivary gland disease, and in one series the recurrence rate for tumours in the parotid, submandibular, and minor salivary glands was 39%, 60%, and 65% respectively (Spiro, 1986). Consequently, adjunctive treatment, particularly with irradiation, is widely practiced (Shidnia *et al.*, 1980). Even so locoregional recurrence remains a persistent problem.

Malignant salivary gland tumours are relatively uncommon forming only 5% of all head and neck malignancies (Rentschler *et al.*, 1977) and thus the experience in any one centre of treating these diseases is limited.

There is little information on the use of chemotherapy for advanced and recurrent malignant tumours as only small studies have been performed on a variety of agents. The most effective drugs are thought to be doxorubicin hydrochloride (dox), Hexamethylmelamine, Cisplatinum, and 5-Fluorouracil (Budd & Groppe, 1983; Vermeer & Pinedo, 1979; Rentschler *et al.*, 1977; Johnson *et al.*, 1964; Alberts *et al.*, 1981; Skibbia *et al.*, 1981; Creagan *et al.*, 1983; Richards & Chambers, 1973; Moore *et al.*, 1968). Dox and 5-Fluorouracil appear to be synergistic.

In the present study the efficacy of the less toxic dox analogue Epirubicin in combination with 5-Fluorouracil is compared with Cisplatinum in the palliation of advanced and recurrent malignant salivary tumours.

### Patients and methods

A Phase II crossover study is reported (Herson, 1984). All patients had recurrent, advanced, malignant tumours of the salivary glands and were unsuitable for surgery or radiotherapy. Patients were randomised by drawing cards from a bag to Cisplatinum only or Epirubicin and 5-Fluorouracil (5FU). If no response occurred after two courses or if the disease progressed the patient was crossed over to the other regimen.

Criteria of response, duration of response, and toxicity were as laid down by Miller *et al.* (1981), and patients were followed up fortnightly.

The number of patients needed for the trial was calculated using Gehan's rule (1961). Fourteen patients were to be admitted initially, a number sufficient to detect a 20% response with a power of 95%. If no response had occurred the trial would have been terminated. In the event 16 patients were admitted to the trial (Tables I and II).

Each tumour was carefully assessed and classified by site and stage (UICC, 1987). The assessment included measuring the perpendicular diameter using calipers; palpation for local neck disease; pharyngeal endoscopy and appropriate radiology. The patients's general condition was classified by the Karnofsky scale (Beahrs *et al.*, 1988) and all patients underwent a complete general physical examination. All patients were seen by a Consultant Physician, as part of their initial assessment and who also advised on management during the administration of chemotherapy.

Investigations included a full blood count and white differential count, serum urea and electrolytes, 24 h creatinine clearance, liver function tests, chest radiography, electrocardiography and a pure tone audiogram.

Specific exclusion criteria included those patients with histology showing squamous cell carcinoma, undifferentiated carcinoma, or lymphoma; patients with impaired renal function characterised by a creatinine clearance of less than 50 ml per min and patients with impaired liver function or significant cardiac disease.

The proposed administration of chemotherapy was discussed with each patient and their relatives by explaining its intended purpose and the possible side effects of the treatment. It was emphasised that the patient had every right to refuse admission to the trial and withdraw at any stage.

The trial was passed by the Royal Liverpool University Hospital Ethics Committee.

### *Dosage and administration*

In order to be treated a patient had to satisfy the following criteria:

(1) A Karnofsky performance status greater than 50.
(2) The patient's white blood count was greater than $4000\,mm^{-3}$ and platelet count greater than $100{,}000\,mm^{-3}$.
(3) Creatinine clearance greater than $50\,ml\,min^{-1}$. The group to be given Cisplatinum were prehydrated with 2 litres of normal saline administered intravenously over 12 h and received one dose of 12.5 g of Mannitol.

The dose of cisplatinum administered was modified according to renal function as follows:

Correspondence: A.S. Jones, Department of Otorhinolaryngology, University of Liverpool, Royal Liverpool Hospital, PO Box 147, Liverpool L69 3BX, UK.
Received 18 June 1992; accepted 15 August 1992.

**Table I** Host factors

| | *Epirubicin + 5-FU* | *Cisplatinum* |
|---|---|---|
| Number | 7 | 9 |
| Age in years (Mean) | 53 | 61 |
| Sex ratio (M:F) | 2.5:1 | 3.5:1 |
| Karnofsky (Median) | 80 | 80 |

**Table II** Tumour factors

| | *Epirubicin + 5-FU* | *Cisplatinum* |
|---|---|---|
| *Histology* | | |
| Adenocarcinoma | 3 | 3 |
| Malignant mixed | 0 | 1 |
| Adenoid cystic carcinoma | 3 | 2 |
| Mucoepidermoid carcinoma | 1 | 2 |
| Acinic cell tumour | 0 | 1 |
| *Site* | | |
| Oropharynx | 0 | 1 |
| Oral cavity | 0 | 3 |
| Submax gland | 2 | 1 |
| Parotid | 5 | 4 |

| *Creatinine clearance ($ml\,min^{-1}$)* | *Cisplatinum ($mg\,m^{-2}$)* |
|---|---|
| >60 | 100 |
| 50–60 | 50 |
| <50 | 0 |

and given over 6–8 h as an intravenous infusion. Post hydration was with 1 l of normal saline over 2 h.

The other group received Epirubicin (75 $mg\,m^{-2}$) given over 6–8 h followed by 5-Fluorouracil (100 $mg\,m^{-2}$) given over 24 h as an intravenous infusion.

The total cumulative doses of Epirubicin did not exceed 700 $mg\,m^{-2}$ and of Cisplatinum, 600 $mg\,m^{-2}$.

*Analysis of the data*

Survival curves were constructed using the method of Kaplan and Meier (1985) and were compared for the two groups using the log rank test (Peto *et al.*, 1977).

Categories of response for the two groups were compared by Fisher's test of exact probability.

### Results

There was no response to treatment in the Epirubicin and 5-Fluorouracil group and only one partial response in those receiving Cisplatinum.

The median survival was 243 days for the Epirubicin/5-FU group and 450 days for the Cisplatinum group. The difference in survival was not significant ($\chi^2 = 0.3$, d.f. = 1).

The results are displayed in Table III.

**Table III** Treatment and outcome

| | *Epirubicin + 5-FU* | *Cisplatinum* |
|---|---|---|
| Median number of courses (Range) | 2 (1–5) | 2 (0–6) |
| Response | | |
| Progressive disease | 5 | 5 |
| Stable disease | 2 | 3 |
| Partial response | 0 | 1 |
| Complete response | 0 | 0 |
| (Fisher's exact test $P = 0.56$) | | |
| Median survival (days) | 243 | 450 |

($\chi^2 = 0.3$, df = 1, N.S.)

### Discussion

Studies concerning malignant salivary gland tumours are rare and have concentrated on small numbers of patients exposed to a variety of chemetherapeutic agents including Methotrexate, 5-FU, dox, Hydroxyurea, C.C.N.U. (1-(2-chlorethyl)-3-cyclohexyl-1-nitrosourea), Vincristine, Cisplatinum, Cyclophosphamide, Hexamethylmelamine, Bleomycin, and Chlorambucil (Budd & Groppe, 1983; Vermeer & Pinedo, 1979; Rentschler *et al.*, 1977; Johnson *et al.*, 1964; Alberts *et al.*, 1981; Skibba *et al.*, 1981; Creagan *et al.*, 1983; Richards & Chambers, 1973; Moore *et al.*, 1968). Some of these studies are case reports and some are trials. The two large chemotherapy series (Rentschler *et al.*, 1977; Tannock & Sutherland, 1980), consist of 36 and 17 patients respectively. Rentschler (1977) reports six patients each giving a partial response with a single agent (dox and Hexamethylamine) and two partial responses with combination chemotherapy from 36 patients treated. The median duration of survival from initiation of chemotherapy was 6 months. Alberts *et al.* (1981) and Creagan *et al.* (1983) in two separate studies using dox, Cyclophosphamide and Cisplatinum both achieved a complete response in two patients, the median duration of this response being 5 months. They also achieved a partial response in three and six patients respectively with a median duration of 6 months in both groups. Other studies (Budd & Groppe, 1983; Vermeer & Pinedo, 1979; Alberts *et al.*, 1981; Tannock & Sutherland, 1980) reported partial responses with median durations of 5 weeks, 5 months, and 12 months. Combination regimens, such as Cylcophosphamide/dox/Cisplatinum (Alberts *et al.*, 1981; Creagan *et al.*, 1983) and Cyclophosphamide/dox/Vincristine (Skibba *et al.*, 1981) show a very high response rate with a median duration ranging from 1 to 7 months.

In the present study there was no response to Epirubicin/5-FU and the median survival was 243 days. There was one partial response in the Cisplatinum group and the median survival was 450 days. The difference was not significant.

### Conclusions

(1) The optimistic view summarised in the Introduction is typical and biased – because only the good results tend to be reported.
(2) Any difference in survival is difficult to analyse because of the well known unpredictable long term behaviour of salivary carcinoma and in particular adenoid cystic carcinoma. Therefore, for all these tumours, response is probably a better criterion than survival unlike squamous cell carcinoma where the converse is true. Furthermore, chemotherapeutic trials which fail to consider the effect of varying histology of malignant salivary gland tumours on response and survival should no longer be considered acceptable.
(3) In this study both chemotherapeutic regimens produced equally disappointing results.
(4) This trial suggests that chemotherapy is unhelpful in the treatment of end-stage salivary malignancy.

The authors are grateful to the North West Cancer Research Campaign for technical assistance, and to Mrs Brenda Cowley who typed the manuscript.

### References

ALBERTS, D.S., MANNING, M.R., COULTHARD, S.W., KOOPMAN, C.F. & HERMAN, T.S. (1981). Adriamycin/Cis-Platinum/Cyclophosphamide combination chemotherapy for advanced carcinoma of the parotid gland. *Cancer*, **47**, 645–648.

AMERICAN JOINT COMMITTEE OF CANCER STAGING AND END RESULT REPORTING (1988). *Manual for Staging Cancer*, 3rd edition. Beahrs, O.H., Henson, D.E., Hutton, R.V. & Myers, M.H. (eds) J.P. Lippincott Company, Philadelphia.

BUDD, G.T. & GROPPE, C.W. (1983). Adenoid cystic carcinoma of the salivary gland: sustained complete response to chemotherapy. *Cancer*, **51**, 589–590.

CREAGAN, E.T., WOODS, J.E., SCHUTT, A.J. & O'FALLON, J.R. (1983). Cyclophosphamide, Adriamycin, and Cis-diamminedichloroplatinum (II) in the treatment of advanced non squamous cell head and neck cancer. *Cancer*, **52**, 2007–2010.

GEHAN, E.A. (1961). The determination of the number of patients required in a preliminary and follow up trial of a new chemotherapeutic agent. *J. Chron. Dis.*, **13**, 346–353.

HERSON, J. (1984). Statistical aspects in the design and analysis of phase II clinical trials. In *Cancer Clinical Trials: Methods and Practice*. Buyse, M.E., Staquet, M.J. & Sylvester, R.J. (eds). Oxford University Press, pp. 239–257.

JOHNSON, R.O., LANGE, R.D., KISKEN, W.A. & CURRERI, A.R. (1964). Infusion of 5-Fluorouracil in cylindroma treatment. *Arch. Otolaryngol.*, **79**, 625–627.

KAPLAN, E.L. & MEIER, P. (1985). Nonparametric estimation from incomplete observations. *J. Am. Stat. Assoc.*, **53**, 457–481.

MILLER, A.B., HOOGSTRATEN, B., STAQUET, M. & WINKLER, A. (1981). Reporting the results of cancer treatment. *Cancer*, **47**, 207–214.

MOORE, G.E., BROSS, I.D. & AUSMAN, R. (1968). Effect of chlorambucil in 374 patients with advanced cancer. *Cancer Chemother. Rep.*, **52**, 661–666.

PETO, R., PIKE, M.C., ARMITAGE, P., BRESLOW, N.E., COX, D.R., HOWARD, S.V., MANTEL, N., MCPHERSON, K., PETO, J. & SMITH, P.G. (1977). Design and analysis of randomized clinical trials requiring prolonged observation of each patient. *Br. J. Cancer*, **35**, 1–39.

RENTSCHLER, R., BURGESS, M.A. & BYERS, R. (1977). Chemotherapy of malignant major salivary gland neoplasms. A 25 year review of M.D. Andersen Hospital experience. *Cancer*, **40**, 619–624.

RICHARDS, G.J. & CHAMBERS, R.G. (1973). Hydroxyurea in the treatment of neoplasms of the head and neck. *Am. J. Surg.*, **126**, 513–518.

SHIDNIA, H., HORNBACK, N.B., HAMAKER, R. & LINGEMAN, R. (1980). Carcinoma of the major salivary glands. *Cancer*, **45**, 693–697.

SKIBBA, J.L., HURLEY, J.D. & RAVELO, H.V. (1981). Complete response of a metastatic adenoid cystic carcinoma of the parotid gland to chemotherapy. *Cancer*, **47**, 2543–2548.

SPIRO, R.H. & SPIRO, J.D. (1989). Cancer of the salivary glands. In *Cancer of the Head and Neck*. Myers, E.N. & Suen, J.Y. (eds). Churchill Livingstone: New York, 2nd edition. pp. 645–668.

SPIRO, R.H. (1986). Salivary neoplasms: overview of a 35 year experience with 2807 patients. *Head and Neck Surg.*, **8**, 177–184.

TANNOCK, I.F. & SUTHERLAND, D.J. (1980). Chemotherapy for adenocystic carcinoma. *Cancer*, **46**, 452–454.

UICC (1987). *Classification of Malignant Tumours* 4th edition. Springer Verlag: Berlin.

VERMEER, R.J. & PINEDO, H.M. (1979). Partial remission of advanced adenoid cystic carcinoma obtained with Adriamycin. *Cancer*, **43**, 1604–1606.